# Disposition Kinetic of Moxifloxacin following Intravenous, Intramuscular, and Subcutaneous Administration in Goats

**DOI:** 10.5402/2011/584342

**Published:** 2011-12-29

**Authors:** Harshad B. Patel, Shailesh K. Mody, Hitesh B. Patel, Vipul A. Patel, Urvesh D. Patel

**Affiliations:** ^1^Department of Pharmacology and Toxicology, College of Veterinary Science and Animal Husbandry, Junagadh Agricultural University, Junagadh 362 001, Gujarat State, India; ^2^Department of Pharmacology and Toxicology, College of Veterinary Science and Animal Husbandry, Sardarkrushinagar Dantiwada Agricultural University, Sardarkrushinagar 385506, Dantiwada, India

## Abstract

The present study was carried out to investigate disposition kinetics of moxifloxacin following single-dose intravenous (i.v.), intramuscular (i.m.), and subcutaneous (s.c.) administration at a dose rate of 5 mg/kg of body weight (b.wt.) in goats. Plasma samples collected after treatments were analyzed for drug concentration using high-performance liquid chromatography (HPLC). After i.v. administration, distribution of the drug was rapid and wide as reflected by high steady-state volume of distribution. Drug elimination was relatively faster with a total body clearance of 0.59 ± 0.03 L/h/kg. Following i.m. injection, the drug has shown the rapid and near-to-complete absorption with bioavailability of 98.20 ± 3.96 per cent. The maximum plasma drug concentration (C_max_) of 1.21 ± 0.04 *μ*g/mL was attained at 1 h (T_max_). The drug was widely distributed as reflected by high apparent volume of distribution. The elimination half-life (*t*_1/2*β*_) of the drug was 6.26 ± 0.08  h. Following s.c. administration, the drug was rapidly absorbed (C_max_: 1.16 ± 0.02 *μ*g/mL; t_max_: 1 h) and slowly eliminated from the body. The elimination half-life and total body clearance (Cl_B_) were 5.61 ± 0.10 h and 0.60 ± 0.03 L/h/kg, respectively. The bioavailability of moxifloxacin following s.c. administration was 90.44 ± 3.96 per cent.

## 1. Introduction

The fluoroquinolones are the fastest growing antibacterial class in terms of global revenue, increasingly being used in dairy animals to treat a wide range of infectious diseases [1]. Moxifloxacin is an 8-methoxy quinolone, derived originally from nalidixic acid that has undergone first a piperazine substitution at position 7 of the naphthyridine nucleus and then fluorination at position 6. The development of moxifloxacin involved a carbon for nitrogen substitution at position 8, as well as a 7-azabicyclo modification, together with insertion of an 8-methoxy side chain [2].

Moxifloxacin has broad spectrum of antibacterial activity. Moxifloxacin is more active against Gram-positive pathogens, with potent activity against *Aerococcus* spp., *Listeria monocytogenes, Micrococcus* spp., *Rhodococcus equi, Stomatococcus mucilaginous,* methicillin-susceptible *Staphylococcus* spp., all beta hemolytic streptococci, viridans streptococci, and *Streptococcus pneumoniae.* It also had good to moderate activity against *Bacillus* spp., *Corynebacterium* spp., *Enterococcus faecalis,* and methicillin-resistant staphylococci. It was the least active of the three agents tested against *Pseudomonas aeruginosa* but had significant activity against other nonfermentative Gram-negative bacilli including *Acinetobacter* spp., *Flavobacterium* spp., *Pseudomonas* spp., *P. aeruginosa,* and *Stenotrophomonas maltophilia *[3]. It is useful for the treatment of community-acquired pneumonia and upper respiratory tract infections [4].

Pharmacokinetics of moxifloxacin have been studied in various animals like lactating goats [5, 6], lactating ewes, [7, 8] camel [9], horse [10], calves [4, 11], rabbit [12], and broiler chicken [13]. The pharmacokinetic data of moxifloxacin in male goat after i.v., i.m., and s.c. administration are not available. Therefore, the present study was conducted to study the pharmacokinetics of moxifloxacin in male goats under tropical environment.

## 2. Materials and Methods

### 2.1. Animals

The experiment was conducted on six healthy male Mehsana goats weighing 25–30 kg. The study protocol was approved by the animal ethics committee. Animals were procured from Livestock Research Station, Sardarkrushinagar Dantiwada Agricultural University, Sardarkrushinagar, Dantiwada, and housed in separate pan at research station. All animals were maintained on standard ration, and water was provided *ad libitum. *The animals were kept for 2-3 weeks prior to the start of experiment for clinical examination to rule out possibility of any disease state.

### 2.2. Experimental Design

The study was conducted by using cross over design. All animals were treated with moxifloxacin at a dose rate of 5 mg/kg b.wt. either i.v., i.m., and s.c. injection. The washout period of fifteen days were observed between different types of treatments during the study to rule out possibility of accumulation of the drug in the body of animals.

The drug was administered via the left jugular vein, gluteus muscle of thigh region, neck region for i.v., i.m., and s.c. administration, respectively. After drug administration through any single route, blood samples (3 mL) were collected from i.v. catheter (Venflon, 22 × 0.9 × 25 mm) fixed into the right jugular vein into 10 mL heparinized centrifuge tube. Following i.v. administration of the drug, blood samples were collected at 0 (prior to treatment), 0.033, 0.166, 0.5, 1, 2, 4, 8, 12, 24, and 36 h after treatment. Following i.m. and s.c administration of the drug, blood samples were collected at 0 (prior to treatment), 0.033, 0.166, 0.5, 1, 2, 4, 8, 12, 24, 36, and 48 h after treatment. Plasma was separated soon after collection by centrifugation at 3000 g for 15 min and transferred to labelled cryovials and stored at −20°C until assayed for moxifloxacin concentration using HPLC procedure.

### 2.3. Moxifloxacin Assay

Plasma concentrations of moxifloxacin were determined by method as described by Siefert et al. [14] using HPLC with fluorescent detector. Plasma samples were precipitated by adding 200 *μ*L of acetonitrile in 200 *μ*L of sample followed by vortex for 5 min and centrifugation for 10 min at 2000 rpm. The supernatant was diluted fourfold with 0.067 M disodium hydrogen phosphate buffer having pH 7.5, transferred to HPLC (AGILLENT, 1100) autosampler vials, and 50 *μ*L of this solution was automatically injected into the HPLC system by auto sampler. Mixture of buffer and acetonitrile (80 : 20) was used as a mobile phase which was pumped (Model: LC-9A) at the rate of 1 mL/min. Chromatographic separation was performed by using C_18_ column (Supelcosil; 250 × 4.6 mm, 5 *μ*) at room temperature. The effluent was monitor using FLD detector (RF-551) with excitation of 296 nm and emission of 504 nm. All chemicals used for assay were of analytical or HPLC grade purchased from S. D. Fine Chem. Ltd, Mumbai, India.

Calibration curves for moxifloxacin in the range 0.005 to 10 *μ*g/mL were prepared with the use of drug-free plasma of nontreated goat. Pooled plasma samples were taken throughout the procedure, and calibration curves were prepared using prepared standard in mobile phase or plasma by plotting areas of peak of drug at the ordinate and the drug concentration at abscissa. Linear regression analysis was used to determine correlation coefficients of calibration curves. The extraction efficiency of the drug under study was measured by comparison of the area of drug from the spiked plasma samples, with area resulting from direct injections of the standards in mobile phase. The interassay precision of the extraction and chromatography procedures was evaluated by processing replicate aliquots of plasma samples (quintuplicate determinations) containing known amounts of the drug on different days. The analytical method used to extract and quantify the plasma concentration of moxifloxacin by chromatographic analysis using the fluorescent detector was validated. The sensitivity of assay method for moxifloxacin was 0.005 *μ*g/mL. The satisfactory interassay and intra-assay precision have been found with the assay. The linearity was observed from 0.005 to 10 *μ*g/mL with mean correlation coefficient (*R* ^2^) > 0.998.

### 2.4. Pharmacokinetic Analysis

Pharmacokinetic parameters were obtained from individual fitted equations as described by Gibaldi and Perrier [15]. The peak concentration (*C*_max⁡_) and time to peak concentration (*T*_max⁡_) were taken directly from the curve. The area under the concentration-time curve (AUC) and the area under the first moment curve (AUMC) were calculated using the linear trapezoidal rule. The mean residence time (MRT) was calculated as AUMC/AUC. The distribution and elimination half-lives were calculated as ln 2 divided by the distribution and elimination rate constants, respectively. The estimated plasma concentration of the drug at zero time (*C*_p(0)_) after its i.v. administration was the sum of the extrapolated zero-time concentrations of the coefficient *A* and *B*. Total body clearance (Cl_B_), apparent volume of distribution (Vd_area_) and volume of distribution at steady state (Vd_ss_) were calculated using following formulas: Cl_B_ = Dose∗*F*/AUC; Vd_area_ = Dose∗*F*/(AUC)(*β*), where for i.v., 100 per cent bioavailability (*F* = 1) was considered and Vd_ss_ = Dose∗AUMC/(AUC)^2^. The absolute bioavailability (*F*) following i.m. and s.c. administration of the drug was calculated as (AUC_i.m.  or  s.c_/AUC_i.v._) × (Dose_i.v._/Dose_i.m.  or  s.c._) × 100. All data were expressed as Mean ± S.E, The mean and S.E. were calculated and the graph was prepared in Microsoft Excel.

### 2.5. PK/PD Integration

The peak plasma drug concentration (*C*_max⁡_) and area under the curve (AUC_0–*∞*_) were applied in the calculation of the predictors of efficacy for concentration-dependent antibiotics: *C*_max⁡_/MIC_90_ and AUC_0–*∞*_/MIC_90_ for i.m. and s.c. administration route. MIC_90_ data of moxifloxacin against caprine bacterial isolates were not worked out till date. To cover most of the susceptible organisms, in this discussion, the MIC_90_ of 0.09 *μ*g/mL of the drug has been taken into consideration [11].

## 3. Results

**Figure 1:**
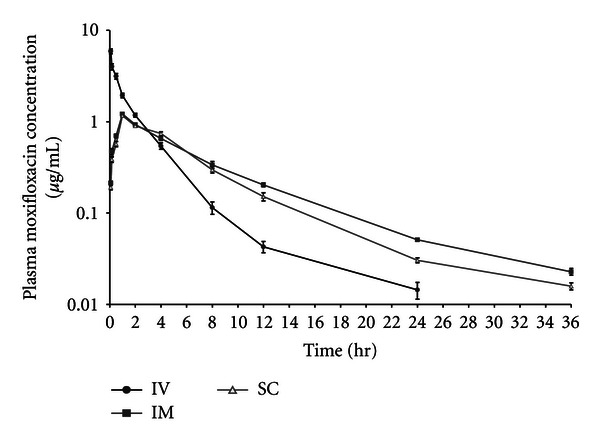
Semilogarithmic plot of moxifloxacin concentrations versus time following single-dose intravenous, intramuscular, and subcutaneous administrations at the rate of 5 mg/kg of body weight in male goat. Each point represents the mean ± S.E. of six animals.

All animals remained in good health throughout the acclimatization and study periods. The drug was distributed according to an open two-compartment model after i.v., i.m., and s.c. administration. The mean (±S.E.) plasma concentrations of moxifloxacin following i.v., i.m., and s.c. administration are plotted in Figure 1. Pharmacokinetic parameters (Mean ± S.E.) estimated after each route of drug administrations are depicted in Table 1.

Following i.v. administration, the apparent volume of distribution (Vd_area_), area under curve (AUC_0–*∞*_), elimination half-life (*t*_1/2*β*_), and total body clearance (Cl_B_) were 3.49 ± 0.32 L/kg, 8.65 ± 0.42 *μ*g·h/mL, 4.12 ± 0.30 h, and 0.59 ± 0.03 L/h/kg, respectively. The drug was absorbed rapidly after i.m and s.c. administration, and the maximum plasma concentration (*C*_max⁡_) of 1.21 ± 0.04 and 1.16 ± 0.02 *μ*g/mL was attained at 1.00 h (*T*_max⁡_) following i.m. and s.c. administration, respectively. After i.m. and s.c. administration, the apparent volume of distribution (Vd_area_), area under curve (AUC_0–*∞*_), elimination half-lives (*t*_1/2*β*_), and total body clearance (Cl_B_) were 5.25 ± 0.30 and 4.74 ± 0.26 L/kg, 8.48 ± 0.18 and 7.75 ± 0.20 *μ*g·h/mL, 6.26 ± 0.09 and 5.61 ± 0.10 h and 0.58 ± 0.03 and 0.60 ± 0.03 L/h/kg, respectively. The bioavailability  (*F*) of the drug following i.m. and s.c. administration were 98.20 ± 3.96 and 90.44 ± 3.96 per cent, respectively.

## 4. Discussion

In the present study, plasma concentration-time profile of the drug following i.v. administration in male goats showed a rapid initial distributive phase, followed by relatively slower elimination phase with an estimated elimination half-life (*t*_1/2*β*_) of 4.12 ± 0.30 h. The drug exhibits a relatively high volume of distribution suggesting an extensive tissue distribution in male goats. The extensive penetration of the drug into various body fluids and tissues owing to its lipid solubility and low plasma protein binding as seen with other members of fluoroquinolones. The body clearance of the drug was 0.59 ± 0.03 L/h/kg which is proximate to the clearance observed in lactating goats [5, 6].

Following i.m. and s.c. administration in goats, the mean peak plasma moxifloxacin concentration (*C*_max⁡_) of 1.21 ± 0.04 and 1.16 ± 0.02 *μ*g/mL were observed as at 1 h (*T*_max⁡_), respectively, which is comparable to the maximum concentrations found in lactating goats [6], sheep [7], ewes, [8] and camel [9] following extravascular administration. The higher volume of distribution of the drug in male goats after all route of administration has been found comparable to that of in lactating goats [6] following extravascular administration indicates a relatively quick and an extensive tissue distribution of the drug in male compared to female animals. The fluoroquinolones due to their high lipid solubility and low plasma protein binding have shown extensive penetration into various body fluids and tissues. Primarily, fluoroquinolones are metabolized in liver and excreted in urine or bile as active drug. The extent of renal elimination varies across the fluoroquinolones. In the present study, total body clearance of the drug following i.v., i.m., and s.c. administration as 0.59 ± 0.03, 0.58 ± 0.03, and 0.60 ± 0.03 L·h/kg, respectively, and are comparable to those (0.43 ± 0.02 and 0.34 ± 0.02 L·h/kg) reported in goats and lactating ewes [6, 8].

The elimination half-life of the drug in male goat has been found longer than observed value in lactating goats [6], ewes [8], and male camel [9] which indicates slower elimination of the drug in male goats compared to lactating goat and sheep. Shorter elimination half-life in lactating goat [6] compared to the value observed in the present study indicates a good penetration of moxifloxacin into the milk. Penetration of moxifloxacin from the blood into milk has been found rapid and extensive in lactating ewes [8]. Longer distribution and elimination half-lives following i.v. administration compared to those observed in lactating ewes [8] is also an indication that lactation state may affect the distribution and elimination of the drug.

The absolute systemic bioavailabilities of the drug in male goats after i.m. and s.c. administration were nearly complete. The values indicate the excellent absorption of the drug from the injection site and the absorption process was relatively fast with absorption half-life (*t*_1/2k_a__) of 0.96 ± 0.19 and 0.82 ± 0.11 h following i.m and s.c., respectively. Higher bioavailability of moxifloxacin have also been found in lactating goats (96.87 ± 10.27 per cent) and ewes (96.35 ± 17.32 per cent) after s.c. administration [6, 8]. In the present study, disposition characteristics following i.m. and s.c. route of administration are near to comparable to each other which indicates that s.c. route of administration can also be utilized in clinical practice.

The pharmacokinetic data following intra- and extra-vascular administration of moxifloxacin in male goats indicates that the drug could have success against susceptible pathogens after parenteral administration. Pharmacokinetic-pharmacodynamic indices have been used to optimize dosing strategies to increase the efficacy of a drug and to minimize the selection of resistant organisms. For a concentration-dependent drug, such as moxifloxacin, successful treatment usually correlates with AUC/MIC_90_ and *C*_max⁡_/MIC_90_, and a high ratio of the *C*_max⁡_/MIC_90_ has also been associated with a lower incidence of the development of resistance [16]. For effective eradication of bacteria and good clinical outcome an AUC/MIC_90_ ratio of >30 for Gram-positive and >100 for Gram-negative organisms has been suggested for fluoroquinolones [17]. A *C*_max⁡_/MIC_90_ of 8–10 would be associated with better clinical results [14]. It is now accepted that high *C*_max⁡_/MIC_90_ value is necessary to avoid bacterial resistance emergence [18]. Moxifloxacin is likely to be effective against bacterial isolates with MIC ≤ 0.09 *μ*g/mL [11]. On the basis of the data, a dosage of 5 mg/kg for moxifloxacin following i.m and s.c. administration in male goats would resulted in AUC*/*MIC90 ratio of 94.22 and 86.11, respectively. However, the values of most important surrogate marker *C*_max⁡_/MIC_90_ were 13.44 and 12.89, respectively, which exceeds the recommended ratios.

In conclusion, lack of local reactions or adverse effects and disposition characteristics of moxifloxacin following i.v., i.m, and s.c. administration may be a indication of a new insight into the strategy for clinical treatment of various bacterial diseases in goat against bacterial isolates with MIC ≤ 0.09 *μ*g/mL.

## Figures and Tables

**Table 1:** Pharmacokinetic parameters (Mean ± S.E.) of moxifloxacin after intravenous, intramuscular, and subcutaneous administration at a dosage of 5 mg/kg of b.wt. in male goat (*n* = 6).

Pharmacokinetic parameters	Units	Intravenous	Intramuscular	Subcutaneous
*t*_1/2*α*_	h	0.74 ± 0.04	—	—
*t*_1/2k_a__	h	—	0.96 ± 0.19	0.82 ± 0.11
*t*_1/2*β*_	h	4.12 ± 0.30	6.26 ± 0.09	5.61 ± 0.10
AUC_0–*∞*_	(*μ*g·h/mL)	8.65 ± 0.42	8.48 ± 0.18	7.75 ± 0.20
AUMC	(*μ*g·h^2^/mL)	24.92 ± 2.13	67.74 ± 1.95	54.42 ± 1.70
Vd_area_	(L/kg)	3.49 ± 0.32	5.25 ± 0.30	4.74 ± 0.26
Vd_ss_	(L/kg)	5.00 ± 0.46	3.39 ± 0.78	3.73 ± 1.28
Cl_B_	(L/kg/h)	0.59 ± 0.03	0.58 ± 0.03	0.60 ± 0.03
MRT	(h)	7.02 ± 0.48	8.00 ± 0.23	7.03 ± 0.13
*C*_max⁡_	(*μ*g/mL)	—	1.21 ± 0.04	1.16 ± 0.02
*T*_max⁡_	(h)	—	1.00	1.00
*F*	(%)	—	98.20 ± 3.96	90.44 ± 3.96

*t*_1/2*α*_: half-life of distribution phases; *t*_1/2k_a__: absorption half-life; *t*_1/2*β*_: elimination half-life; AUC_(0–*∞*)_: area under the curve from zero to infinity; AUMC: area under first of moment curve; Vd_area_: apparent volume of distribution; Vd_ss_: volume of distribution at steady state; Cl_B_: total body clearance; MRT: mean residence time; *C*_max⁡_: maximum drug concentration; *T*_max⁡_: time to peak plasma drug concentration; *F*: bioavailability.
